# Supplementary material for: Beta-band activity in medial prefrontal cortex predicts source memory encoding and retrieval accuracy
Source: Sci Rep. 2019 May 2;9:6814. doi: 10.1038/s41598-019-43291-7 (PMC6497659; doi:10.1038/s41598-019-43291-7)
Supplement: Supplementary file 1 — Supplementary Figures [file 41598_2019_43291_MOESM1_ESM.docx]

**Beta-band activity in medial prefrontal cortex predicts source memory encoding and retrieval accuracy**

**Authors: Karuna Subramaniam^a*^, Leighton B.N. Hinkley^b^, Danielle Mizuiri^b^, Hardik Kothare^b^, Chang Cai^b^, Coleman Garrett^b^, Anne Findlay^b^, John F. Houde^b^ and Srikantan S. Nagarajan^b^**

^a^ Department of Psychiatry, University of California, San Francisco, CA-94143, USA

^b^ Department of Radiology and Biomedical Imaging, University of California, San Francisco, CA-94143, USA

**Key words: self-agency, reality monitoring, medial prefrontal cortex, encoding self-generated thoughts, retrieving self-generated thoughts**

**Declarations of interest: none**

**Corresponding Author and Reprint Requests to be sent to:**

Karuna Subramaniam, Ph.D.

Department of Psychiatry, University of California, San Francisco

513 Parnassus Avenue, HSE604

San Francisco, CA 94143

Email: karuna[.subramaniam@ucsf.edu](mailto:subramaniam@ucsf.edu)

Phone: 1-415-476-6888

Fax: 1-415-502-4302

Running Title: Self-agency during reality monitoring


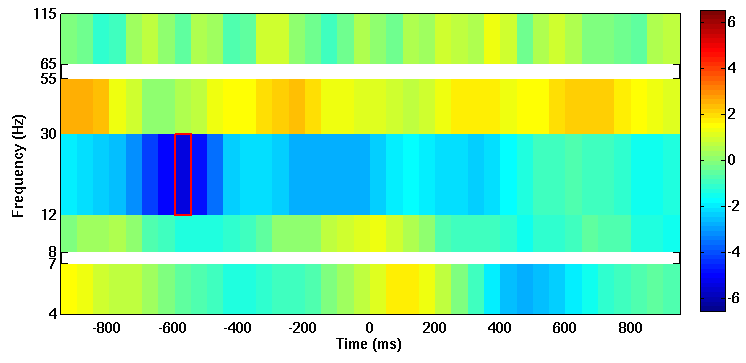


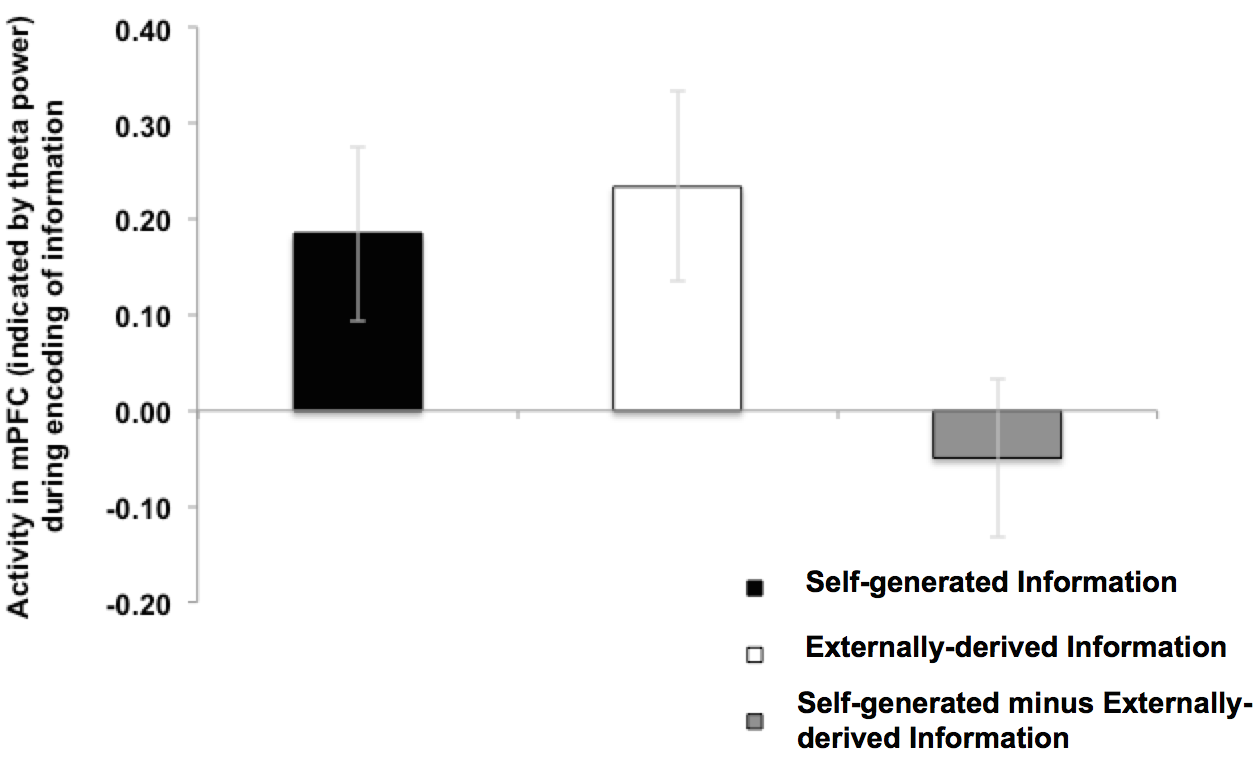

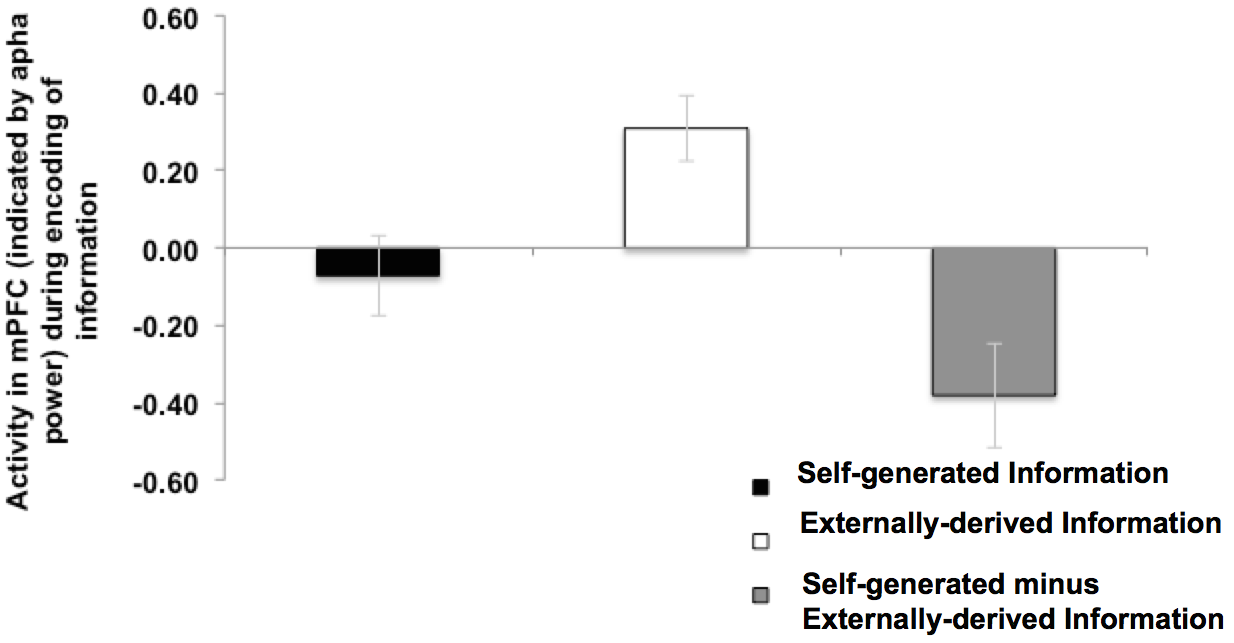

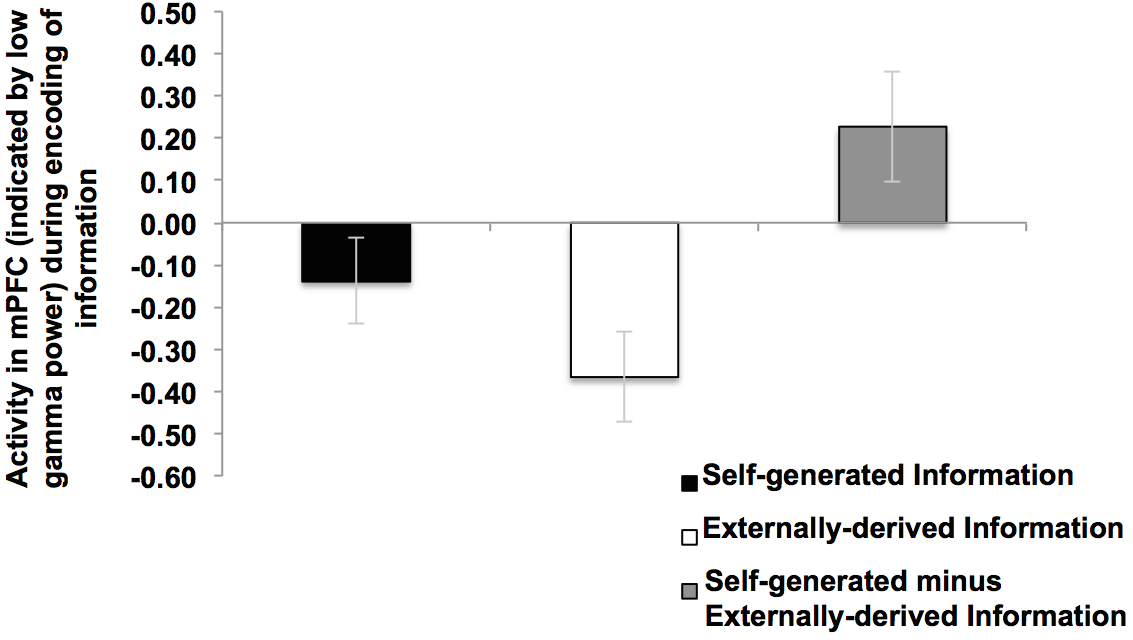

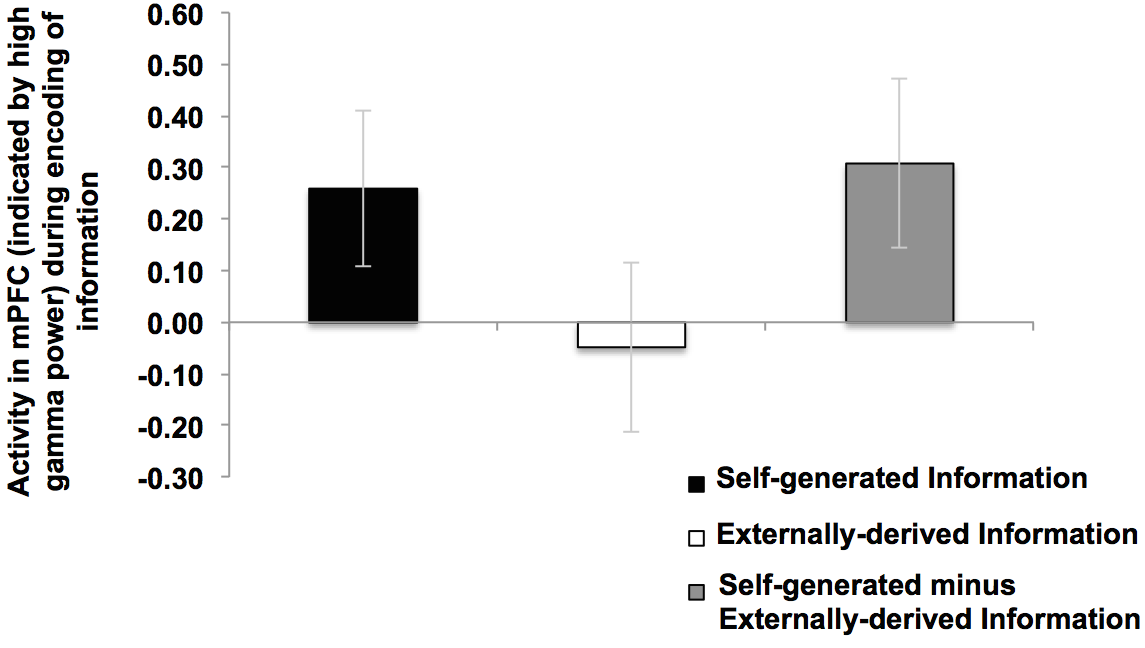


**Supplementary Figure 1. Self-generated Information Encoding Time Windows:** Group analyses of changes in theta (4-7Hz), alpha (8-12Hz), low gamma (30-55Hz) and high gamma (65-115Hz) oscillatory power, indicating non-significant changes in mPFC activity during self-generated encoding (p>.01, uncorrected) between -700 to -500ms prior to vocalization onset (0ms). Bar charts illustrate beta weights averaged across all participants for each condition for each band (theta, alpha, low and high gamma), centered at the peak of mPFC activity increase which was found only in beta band (x,y,z = -15,40,0, see Figure 2) during encoding of self-generated vs. externally-derived information.


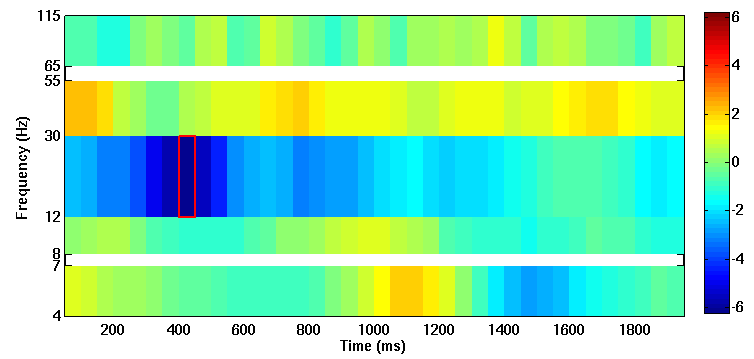


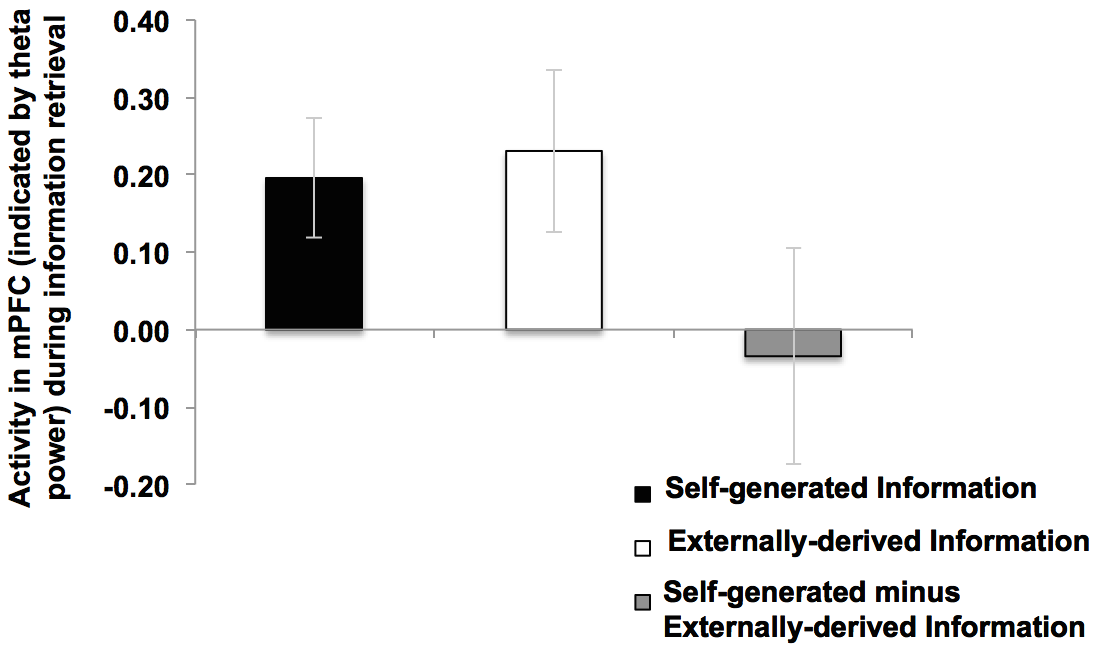

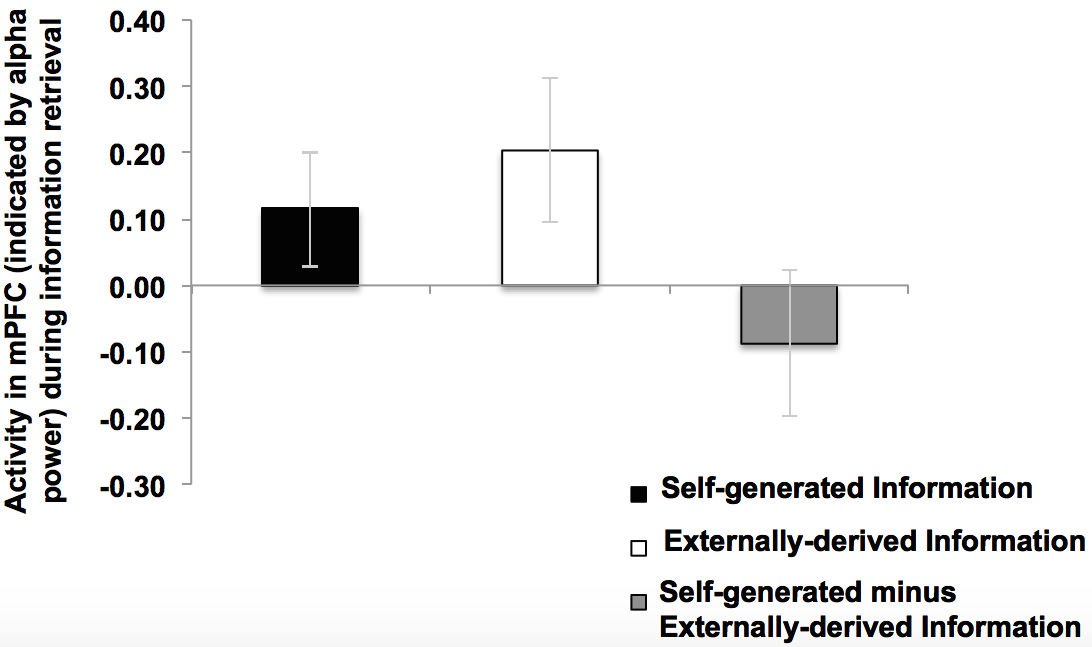

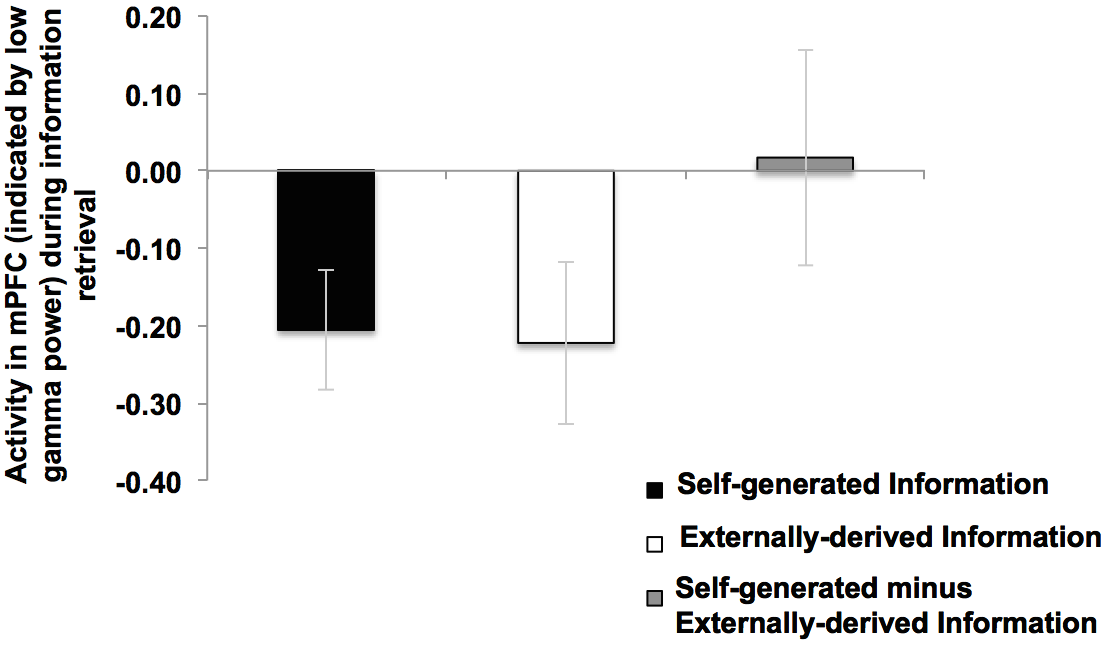

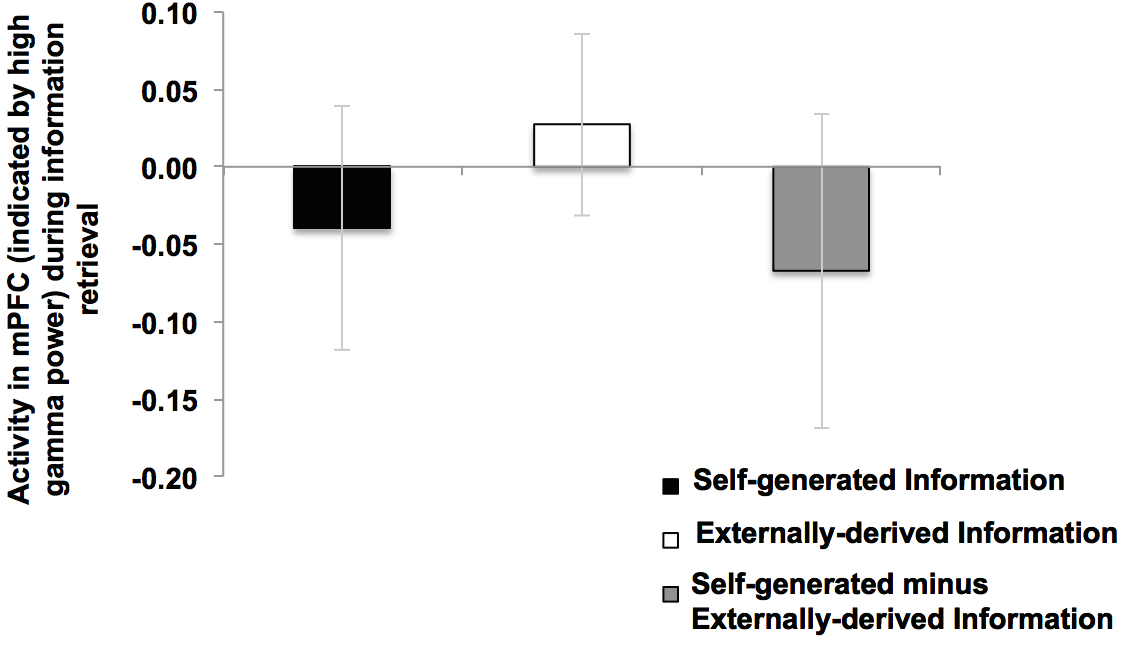


**Supplementary Figure 2.** **Self-generated Information Retrieval Time Windows:** Group analyses of changes in theta (4-7Hz), alpha (8-12Hz), low gamma (30-55Hz) and high gamma (65-115Hz) oscillatory power, indicating non-significant changes in mPFC activity during self-generated retrieval (p>.01, uncorrected) between 300 to 500ms after stimulus onset (0ms). Bar charts illustrate beta weights averaged across all participants for each condition for each band (theta, alpha, low and high gamma), centered at the peak of mPFC activity increase which was found only in beta band (x,y,z = -15,40,0, see Figure 3) during retrieval of self-generated vs. externally-derived information.


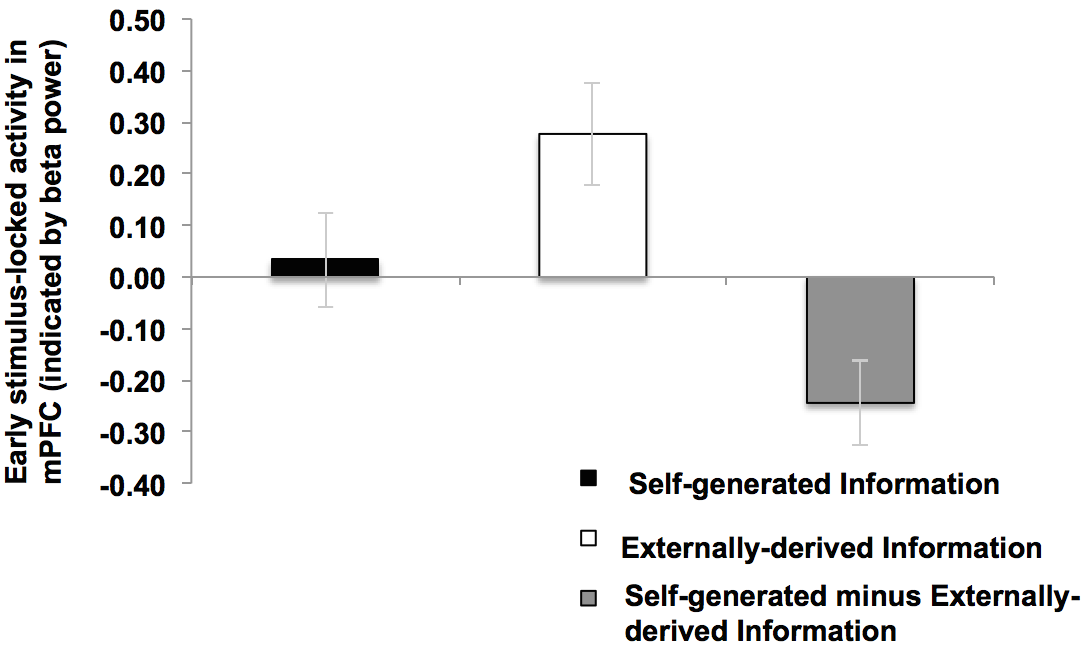

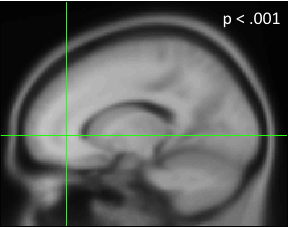

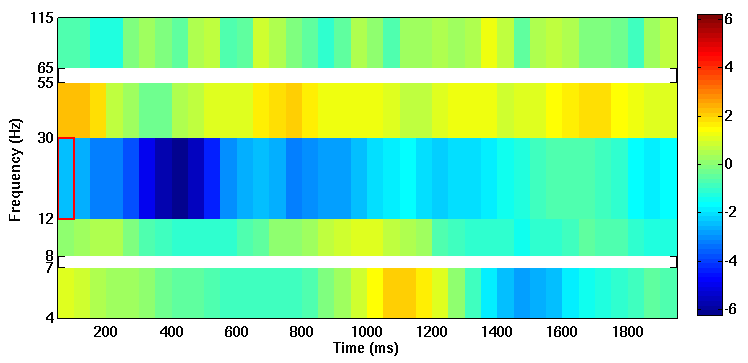


**Supplementary Figure 3.** Stimulus-locked (0ms=word-pair onset) group analyses of changes in beta (12-30Hz), indicating non-significant changes in mPFC activity (p>.001, uncorrected) between 0-200ms after stimulus onset. Brain images yoked to stimulus presentation, do not show mPFC activation thresholded at p < 0.001, cross-hairs centered at the peak of mPFC activity (x,y,z = -15,40,0). This lack of mPFC activity observed in the first 200ms after stimuli onset indicates that mPFC activity cannot be attributed to general stimuli properties of the self-generated condition.
